# Japanese Community Pharmacists’ Dispensing Influences Medicine Price Reduction more than Prescription Numbers 

**DOI:** 10.5539/gjhs.v8n9p20

**Published:** 2015-10-16

**Authors:** Masayuki Yokoi, Takao Tashiro

**Affiliations:** 1 School of Graduate Studies, The Open University of Japan, Chiba, Japan; 2 Pascal Pharmacy, Shiga, Japan

**Keywords:** costs, medicine, number, regression, separation system

## Abstract

This study examined the economic efficiency of the separation of prescription and dispensation medicines between doctors in medical institutions and pharmacists in pharmacies. *The separation system* in Japanese prefectures was examined with publicly available data (Ministry of Health, Labour and Welfare, 2012–2014; retrieved from http://www.mhlw.go.jp/topics/medias/year). We investigated whether the *separation system* reduces the number of medicines or the medication cost of a prescription because of separating the economic management between prescribing and dispensing and the effect of mutual observation between doctors and pharmacists. It is optional for Japanese medical institutions to participate in *the separation system*. Consequently, the spreading rate of *the separation system* in each administrative district is highly variable. We examined the *separation system* effect using the National Healthcare Insurance data for three years, 2012–2014. We tested whether the *separation system* ratio for each prefecture was significantly correlated to the medication price or the number of medicines on a prescription. If spreading the *separation system* influenced the price of prescribed daily medications or the number of medicines, the correlation would be significant. As a result, the medication price was significantly negatively correlated with the *separation system* ratio, but the number of medicines was not significant. Therefore, the *separation system* was effective in reducing daily medication cost but had little influence on reducing the number of daily medicines. This was observed over three years in Japan.

## 1. Introduction

A common problem for the advanced nations moving into aged societies is yearly medical cost increases. Aged people have to live with sickness and many live with multiple chronic conditions and disabilities that erode the quality of life. The consequences are also costly for society. In the United States, the most costly 5% of Medicare beneficiaries account for approximately 50% of Medicare’s expenditures (Nikolich-Žugich et al., 2015). Each national government tries to solve this difficult problem. The Japanese government also has been engaged in this problem. It adopted policies to reduce medication costs. Although prescribed medication was mainly dispensed by the prescribing doctor for many years in Japan, it became an option in the 1960s to separate prescribing and dispensing medicines between doctors in medical institutions and pharmacists in pharmacies (hereafter referred to as the *separation system*) like various countries of Europe and the U.S.A. The government adopted the *separation system* to contribute to the efficiency of medication costs because it avoided the practice of doctors’ prescribing medicines for economic motives. The *separation system* is characterized by the prescribing doctor deciding what medications patients buy but not selling them; the pharmacist does not decide what patients purchase but dispenses and sells them. The division of prescription and dispensation avoids the buying of medications by patients due to seller circumstances. Therefore, the *separation system* was considered effective in reducing medication expenses. In this way, medication costs were initially expected to be controlled and reduced by introducing *the separation system* in Japan (Nakamura, Nagaki, Iizuka, & Fujii, 1989). However, until now, few studies have investigated this system.

It have been rarely researched with quantitative data whether *the separation system* is effective for reducing medicine costs, as the methodology to compare the objects accurately is very difficult. It has been unclear whether *the separation system* contributes to doctors’ proper prescription of medicines or reduction of medicine costs (Nakamura et al., 1989).

In one of the few studies, changes in medicine charges due to the *separation system* were compared between Ota Ward in Tokyo, Haibara Ward in Shizuoka prefecture, Ueda City in Nagano prefecture, and Wakamatsu Ward in Kitakyushu City, Fukuoka prefecture. Although medicine charges increased by 30% in Haibara Ward due to the *separation system*, they decreased by 10–20% in the other three districts (Watanabe, 1996). As this result was inconsistent, restrictive in area, and completed over 20 years ago, their findings did not provide robust support for the conception that *the separation system* contributes to reducing medicine costs.

Another study in more recent years, although a relatively small sample of 24 medical institutions including small hospitals and clinics, compared medicine charges before and after the switch to dispensing medicines outside community pharmacies from inside medical institutions (Kinoshita et al. 2004). However, there was no conclusion about whether the medicine costs under *the separation system* were lower than when medicine was prescribed and dispensed at medical institutions.

Thus, reports have differed about whether the *separation system* has been effective in reducing medication costs since the 1960s. Most economic evaluations of clinical pharmacy interventions suffered from a number of methodological limitations including the absence of a control group (De Rijdt, Willems, & Simoen, 2008). The economic effectiveness of *the separation system* has not been quantitatively investigated as it is difficult to accurately compare the objects under study. In particular, a double blind test of the *separation system* is impossible since the experience contents must be known by doctors, patients, and pharmacists. Despite these difficulties, the *separation system* spread rapidly in the 1990s in Japan and is now present in 70% of healthcare facilities in 2015.

Previously, we examined the influence of *the* *separation system* on medication expenditures in Japan. Multiple regression analysis of the complete Japanese public health insurance database showed that the spread of *the* *separation system* and the rate of replacing brand names with generic medications had a significant negative partial correlation with daily internal medicine costs (Yokoi, & Tashiro, 2014). This study was the first to show that expanding the *separation system* was effective in reducing medication costs. An effect was identified because *the* *separation system* is optional in Japan. As Japanese doctors in hospitals or clinics can choose whether to participate in *the separation system*, the daily medication expense data under various spreading rates and consequently the effect of the *separation system* on medicine expenditure could be calculated. Thus, it was revealed that promoting *the separation system* was as effective in reducing internal medicine costs as using the generic medicines.

Following this, we examined the influence of the *separation system* on the total medicine costs including injection, medical devices, external medicines, and additionally each item cost that may be prescribed under the Japanese pharmaceutical affairs law. The spreading *separation system* was significantly negatively correlated with medication costs except for external medicines. Of those, light-pain-killer patches can be prescribed by Japanese law under public health insurance. Moreover, they account for 70% of the external medicine market by the legal price using public health insurance. As professional medical knowledge is not needed for the selection and purchase of light-pain-killer patches, the *separation system* did not appear to affect it. The *separation system* in Japan affects medication costs by removing seller motives. On the other hand, it loses effects when buyers, patients, can take the lead to purchase medicines (Yokoi, & Tashiro, 2015).

Although these studies did not strictly clarify why these medicine expenses decreased with the increased spreading rate, they provided valuable evidence for the economic effect of *the* *separation system*. Therefore, we investigated whether the *separation system* reduces the number of medications on the prescription or the costs of the medications themselves from separating the economic management between prescribing and dispensing and the effects of mutual observation between doctors and pharmacists.

## 2. Method

A universal national health insurance system covers Japan. In brief, all Japanese people must be insured by a specific public health insurance system under Japanese law with exceptions applied to welfare for livelihood protection. There are two organizations providing public health insurance in Japan. One is managed by the Social Insurance Payment Fund for people of private enterprises and government employees. The other is by National Health Insurance for independent businessmen, farmers, artists, unemployed people, and retirees. Therefore, we can access all Japanese medical insurance data if we combine them. The Japanese Ministry of Health, Labour and Welfare (MHLW) generally provides the combined insurance data. We can access the data of every fiscal year for community pharmacies on the MHLW website (MHLW, 2015).

As a result, we can study the medical costs of all of Japan. Importantly, due to repeated changes in the Japanese insurance system, it is difficult to compare the precise economic effect of each medical institution’s adoption of *the separation system*. Additionally, the *separation system* was first adopted by most key medical institutions that were large scale and had the leading medical technology in each district. The current ratio of the *separation system* in Japan is about 70%.

In this study, we examined the *separation system’s* effects on prescriptions for reducing medication costs by analyzing public data from each prefecture in Japan from 2012 to 2014. Japanese prefectures are one of the administrative compartments that divide Japan into 47 districts. Although the discretionary power is not as strong as the state governments in the U.S.A., there are local governments in Japanese prefectures. Each of them can establish their own regulations and has its own budget. Therefore, various statistical data in Japan are generally gathered by each prefecture. Using the public national health insurance database (MEDIAS) of the Japanese MHLW, we obtained the average data of the daily cost of a medication per prescription and daily number of medicines per prescription from each prefecture. MHLW makes all Japanese MEDIAS health insurance data public on its website (MHLW, 2015) including both the Social Insurance Payment Fund and the National Health Insurance. Data was analyzed to provide a clear understanding of the effective causes of medication costs. We examined why expanding the *separation system* reduced medication costs as follows.

### 2.1 Total Medicine Cost Divided into Medication Price and Number of the Prescribed Medicines

Daily medicine cost per prescription was determined by:

*TC = PM × NM* (1)

TC: the total daily cost of medicine

PM: the price of daily medicine

NM: the number of prescribed daily medicines

### 2.2 The average prices of medicine on a prescription and the separation system ratio (%)

We examined *the separation system* effect for the reduced price of each medicine (PM in equation (1)) or reducing the numbers of medicines (NM in Equation (1)) on a prescription. We tested whether the *separation system* ratio (%) of each prefecture was significantly correlated with the average price of the medicines on a prescription. If spreading the *separation system* influenced the price of daily prescribed medicines, the correlation would be significant.

#### 2.2.1 Dependent Variables: the Average Prices of a Medicine on a Prescription

We obtained 2012–2014 fiscal data on average medicine expenses and dosage days per prescription for each prefecture (MHLW, 2012–2014) in the same way as previously (Yokoi, & Tashiro, 2014).

We then directly obtained from the database both the average daily price of a medication per prescription (PM) and the number of daily medicines on a prescription (NM) for each prefecture. Table 1 shows the statistical parameters of the average daily price of a medication as dependent variables used in the data analysis from the 47 Japanese prefectures over three years. Namely, the number of observations is the number of Japanese prefectures. Each expense is per prescription.

**Table 1:** Statistical data daily medication price (USD 1 cent = 1 yen)

Fiscal year	2012	2013	2014
N	47	47	47
Mean	84.2	86.9	85.2
Maximum	90.6	94.4	93.4
Minimum	77.3	79.7	77.4
Standard deviation	3.36	3.45	3.67

#### 2.2.2 Independent Variable: Spreading Rate of *the Separation System*

The independent variable was the spreading rate of *the separation system*. We obtained 2012–2014 fiscal data of the spreading rate of *the* *separation system* among prefectures in Japan (Japan Pharmaceutical Association [JPA]) in the same way as our previous article. The spreading rate of *the separation system* estimated by the JPA is calculated by statistics provided by the annual reports of the Insurance Association for Private Organizations and National Health Insurance. An estimated level of overall prescription numbers anticipated in Japan was calculated by JPA as mentioned in a previous study (Yokoi, & Tashiro, 2015).

The JPA provided the spreading rate of the *separating system* for each Japanese prefecture on their website (JPA, 2012–2014). These data are claimed as the formal spreading rate of the *separation system* in Japan. We used this data as independent variables for single linear regression analysis.

Table 2 shows the statistical parameters of the spreading rate of *the separating system* of each Japanese prefecture as independent variables. The number of observations is the number of Japanese prefectures.

**Table 2:** Statistical data of the spreading rate (%) of the *separating system*

Fiscal year	2012	2013	2014
N	47	47	47
Mean	64.3	65.4	67.2
Maximum	82.7	82.8	84.2
Minimum	37.8	40.7	45.0
Standard deviation	10.1	9.76	9.30

### 2.3 Number of Medicines on a Prescription and the Separation System Ratio (%)

We then examined the *separation system* effect on reducing the number of medicines (NM in equation (1)) on a prescription. We tested whether *the separation system* ratio (%) of each prefecture had a significant correlation with the average number of medicines on a prescription. If spreading the *separation system* influenced the number of prescribed daily medicines, the correlation would be significant.

#### 2.3.1 Dependent Variables: The Number of Daily Prescribed Medicines

We obtained 2012–2014 fiscal data of the average number of daily medicines per prescription for each prefecture (MHLW, 2012–2014). Table 3 shows the statistical parameters of the numbers of daily medicines as dependent variables used in the data analysis from the 47 Japanese prefectures.

**Table 3:** Statistical data of the numbers of daily medicine

Fiscal year	2012	2013	2014
N	47	47	47
Mean	2.95	2.95	2.95
Maximum	3.25	3.23	3.29
Minimum	2.67	2.67	2.66
Standard deviation	0.14	0.14	0.14

#### 2.3.2 Independent Variable: Spreading Rate (%) of the Separation System

The independent variable was the spreading rate (%) of the *separation system*; therefore, it was same as Table 2.

### 2.4 Data Analysis

We calculated the regression coefficient, constant, single regression coefficient (R), and P-value using Excel. Each data point with the exception of the number of prefectures was considered to three significant figures. The number of the prefectures was entered as an integer.

## 3. Results

### 3.1 Correlation between Medicine Price and the Separation System Ratio (%)

Table 4 designates the results of single correlation analysis for the average price of a medicine on daily medical cost. This table shows each single correlation coefficient *R* and P-values from 2012 to 2014.

**Table 4:** Single correlation coefficient and probability

Fiscal year	Regression coefficient	Constant	Single regression coefficient (*R*)	P-value
2012	-0.149	93.7	-0.451	< 0.01
2013	-0.150	96.7	-0.432	< 0.01
2014	-0.194	98.3	-0.490	< 0.001

The results revealed a significant correlation between the spreading rate (%) of the *separation system* and the daily price of medication per prescription. Each “-” regression coefficient is negative. Thus, the more the *separation system* spread, the lower the medication price on a prescription.

### 3.2 Correlation between the Numbers of Daily Medicines and the Separation System Ratio (%)

Table 5 designates the results of single correlation analysis for the average number of daily medicines and the rate of *the separation system*. This table shows each single correlation coefficient *R* and P-value from 2012 to 2014.

**Table 5:** Single correlation coefficient and probability

Fiscal year	Regression coefficient	Constant	Single regression coefficient (*R*)	P-value
2012	-0.0032	3.15	-0.231	0.118
2013	-0.0032	3.15	-0.225	0.128
2014	-0.0040	3.22	-0.257	0.082

There was no significant correlation between the spreading rate of the *separation system* (%) and the number of daily medicines per prescription. The correlation of the numbers of medicines with the *separation system* is much weaker than that of the daily price of medicine with the *separation system*.

## 4. Discussion

We found a significant correlation between the spreading rate of *the separation system* and the daily price of medicine per prescription. This means that the *separation system* reduced the medication price on a prescription. Thus, spreading the *separation system* makes prescribing doctors refrain from use of high price medicines. In the Japanese public insurance system, medicine price is determined by MHLW. Therefore, Japanese medicine prices are officially fixed. Each price of specific medicine is the same price at every medical institution and pharmacy. Consequently, the reason that *the separation system* reduced the medication price is the effect of community pharmacists replacing bland medicines with generic medicines, or to hold doctors prescribing high price medicines. We cannot determine which occurred in this study.

On the other hand, a significant correlation between the spreading rate of *the separation system* and the number of daily medicines per prescription was not observed. The *separation system* did not influence reduction of the number of medicines per prescription. If the *separation system* influenced doctors to improve prescribing, the number of daily medicines per prescription would probably reduce because of removing unnecessary or harmful medicines. However, we observed no evidence for such an influence in this study.

Community pharmacists in Japan are thought to intervene not on the number of prescribed medicines but on the price in some fashion. It could not be determined how prescriptions were influenced in this study. It requires further research.

### 4.1 Limitations of this Study

Although we found that the spreading rate was significantly negatively correlated with daily medicine costs per prescription and not significantly correlated with the number of medicines, the particular causal relationships remain obscure. While there are some possible causes, they are only hypotheses and this study was not able to verify them. For a clear explanation, we must research the actions of doctors, pharmacists, and patients. This remains for further research.

## 5. Conclusions

The *separation system* was efficient in reducing daily medicine cost and had little influence on reducing the number of daily medicines. This was observed over three years in Japan.
